# Do clinical interview transcripts generated by speech recognition software improve clinical reasoning performance in mock patient encounters? A prospective observational study

**DOI:** 10.1186/s12909-023-04246-9

**Published:** 2023-04-21

**Authors:** Kiyoshi Shikino, Tomoko Tsukamoto, Kazutaka Noda, Yoshiyuki Ohira, Daiki Yokokawa, Yuta Hirose, Eri Sato, Tsutomu Mito, Takahiro Ota, Yota Katsuyama, Takanori Uehara, Masatomi Ikusaka

**Affiliations:** 1 Department of General Medicine, Chiba University Hospital, 1-8-1, Inohana, Chuo-Ku, Chiba City, Chiba Pref Japan; 2 Division of General Internal Medicine, Department of Internal Medicine, St. Marianna University School of Medicine Hospital, Kawasaki, Japan

**Keywords:** Feedback, Medical interview, Mini-CEX, SRS

## Abstract

**Background:**

To investigate whether speech recognition software for generating interview transcripts can provide more specific and precise feedback for evaluating medical interviews.

**Methods:**

The effects of the two feedback methods on student performance in medical interviews were compared using a prospective observational trial. Seventy-nine medical students in a clinical clerkship were assigned to receive either speech-recognition feedback (*n* = 39; *SRS feedback* group) or voice-recording feedback (*n* = 40; *IC recorder feedback* group). All students’ medical interviewing skills during mock patient encounters were assessed twice, first using a mini-clinical evaluation exercise (mini-CEX) and then a checklist. Medical students then made the most appropriate diagnoses based on medical interviews. The diagnostic accuracy, mini-CEX, and checklist scores of the two groups were compared.

**Results:**

According to the study results, the mean diagnostic accuracy rate (*SRS feedback* group:1st mock 51.3%, 2nd mock 89.7%; *IC recorder feedback* group, 57.5%–67.5%; F(1, 77) = 4.0; *p* = 0.049), mini-CEX scores for overall clinical competence (*SRS feedback* group: 1st mock 5.2 ± 1.1, 2nd mock 7.4 ± 0.9; *IC recorder feedback* group: 1st mock 5.6 ± 1.4, 2nd mock 6.1 ± 1.2; F(1, 77) = 35.7; *p* < 0.001), and checklist scores for clinical performance (*SRS feedback* group: 1st mock 12.2 ± 2.4, 2nd mock 16.1 ± 1.7; *IC recorder feedback* group: 1st mock 13.1 ± 2.5, 2nd mock 13.8 ± 2.6; F(1, 77) = 26.1; *p* < 0.001) were higher with speech recognition-based feedback.

**Conclusions:**

Speech-recognition-based feedback leads to higher diagnostic accuracy rates and higher mini-CEX and checklist scores.

**Trial registration:**

This study was registered in the Japan Registry of Clinical Trials on June 14, 2022. Due to our misunderstanding of the trial registration requirements, we registered the trial retrospectively. This study was registered in the Japan Registry of Clinical Trials on 7/7/2022 (Clinical trial registration number: jRCT1030220188).

**Supplementary Information:**

The online version contains supplementary material available at 10.1186/s12909-023-04246-9.

## Background

Studies show that 70%–90% of medical outpatients are diagnosed based on their medical history; [1, 2] thus, medical history contributes greatly to diagnosis. However, making a diagnosis from a medical history depends on the medical practitioners’ acquisition of inference skills and medical history-taking skills [3]. Medical interviews conducted with simulated patients are important as they expand the knowledge gained during undergraduate studies and actual medical practice [4]. Additionally, medical interviews effectively improve the practitioners’ clinical reasoning abilities and medical history-taking skills.

These skills should be further supplemented by effective learning strategies such as appropriate feedback-based guidance from instructors, empowering students to make their own diagnoses and correct or improve their attempts [5, 6]. Supervising instructors often provide feedback immediately after medical interviews. However, as this type of guidance depends on the instructors’ short-term memory, the clarity, concreteness, and uniformity of instruction might be reduced. Although feedback based on recorded medical interviews can address these issues, checking the recorded content and extracting problems can be time-consuming and place a greater burden on instructors.

Thus, our study used a speech recognition system (SRS) to obtain data from medical interviews conducted in Japanese and to provide feedback. Since the subjects’ native language in this study was Japanese, the SRS for Japanese was used in this study. The SRS can recognize and transcribe medical interviews accurately and quickly, thus enabling clear, specific, and effective feedback to be provided easily. As SRS can instantly and accurately transcribe verbal interactions, it enables the review of conversational exchanges in text format and, thus, a detailed analysis of doctor–patient conversations. It also greatly improves the turnaround times for reports compared to remote transcription and allows for immediate and better control of report editing compared to traditional paper markup or asynchronous transcription modification methods [7–12]. Physicians and nurses increasingly use SRS for documentation [13]. It has also been used for recording radiology reports and improving the overall examination turnaround and report production time [7, 8, 12]. It can also replace typing and thus reduce user fatigue [14]. SRS has also reduced the loss of information in nursing reports and increased the quality of nursing documentation through direct and on-time data recording [15, 16]. Although several useful data sources provide clinical performance feedback, we introduce interview transcripts generated using SRS software as a more specific feedback source for evaluating medical history-taking skills.

Our study aimed to compare the conventional medical interview feedback method with the SR-based feedback method. We investigated whether this method was superior to a feedback method based solely on voice recordings for clinical skill training. Medical interview feedback should be based on specific learner activities [17]. Several methods can be used to record specific activities, such as video and voice recordings [18], but our study considered SRS and voice recordings to investigate feedback methods limited to verbal information. We believe that using SRS-based medical interview feedback in text format requires less time and is more effective in education than using recorded medical interview-based feedback.

## Methods

### Study design and setting

**Fig. 1:**
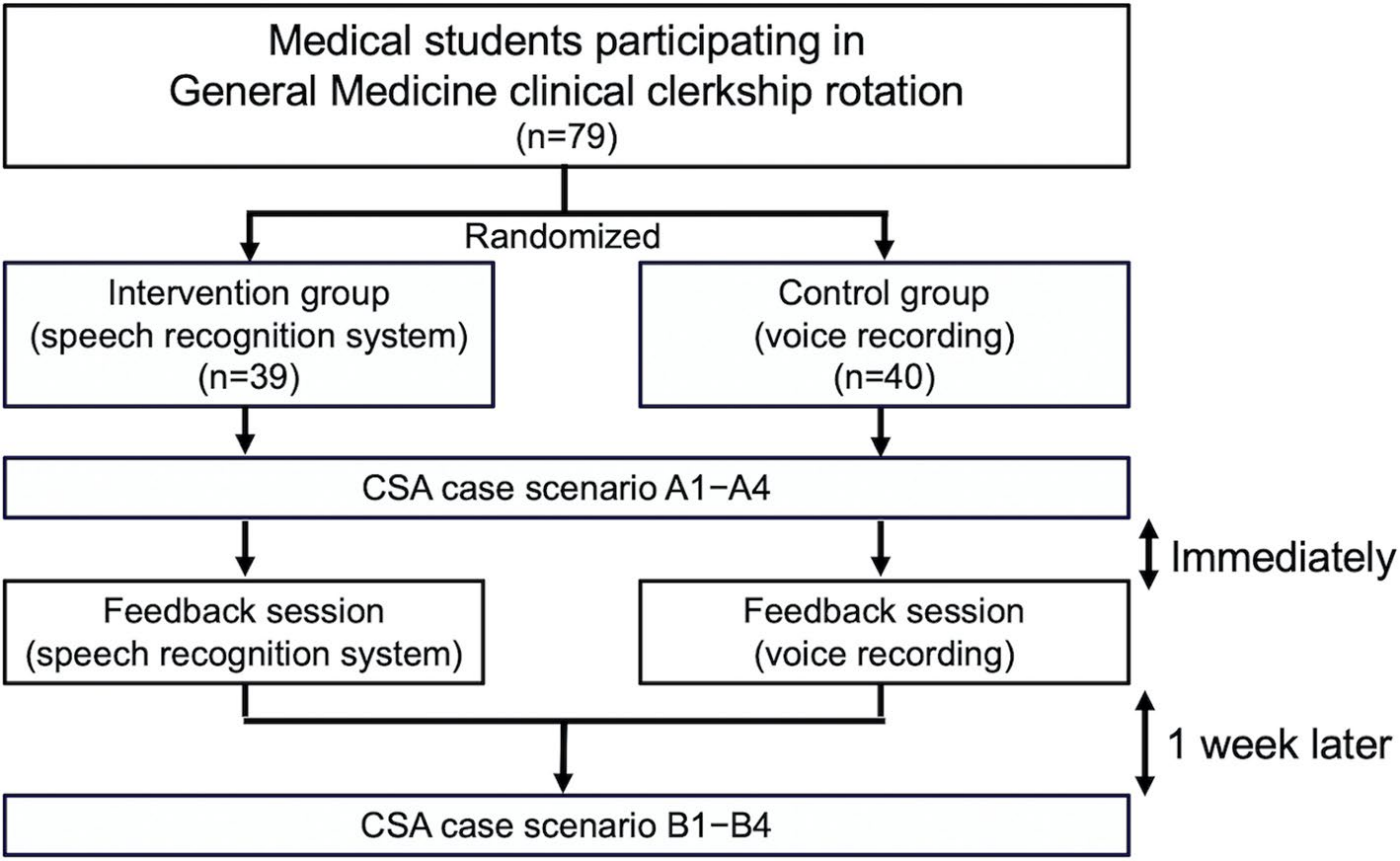
Outline of the study

This prospective observational study compared the effects of two practical feedback methods on medical students’ performance in mock patient encounters (Fig. 1). Seventy-nine fifth-year medical students pursuing a general medicine clinical clerkship were included in the study, which was conducted from June 2016 to January 2017. Clinical clerkship begins with a 2-week training program for a total of 5–6 medical students in an outpatient setting. Medical students were divided into two groups–an SRS feedback group and a voice recording feedback group–using a simple randomization method in Excel 2010 (Microsoft Corp.). This study followed the Consolidated Standards of Reporting Trials (STROBE) reporting guidelines.

### Mock patient encounter and feedback

#### First mock patient encounter

Each student was assigned one of four clinical encounter cases (A1–A4) and asked to make a differential diagnosis based on the case history and physical examination findings. A trained simulated patient provided students with a case history in response to questions based on the case scenario. The student then asked the simulated patient about the physical examinations considered necessary, and the simulated patient orally provided the student with the physical examination findings. Medical students were required to make the most appropriate diagnosis based on their case history and physical examination findings. This encounter took place during the first week of the clinical clerkship. Six simulated patients were asked to play the role of a patient. To minimize the problems related to role-playing and variations in feedback methods, we introduced several faculty developments and standardized the content.

After the mock simulated patient encounters, the medical student received one of two feedback methods–the SRS or IC recorder feedback method–from a faculty member who directly observed the medical interview and physical examination.

#### Feedback methods (educational interventions)

**Fig. 2:**
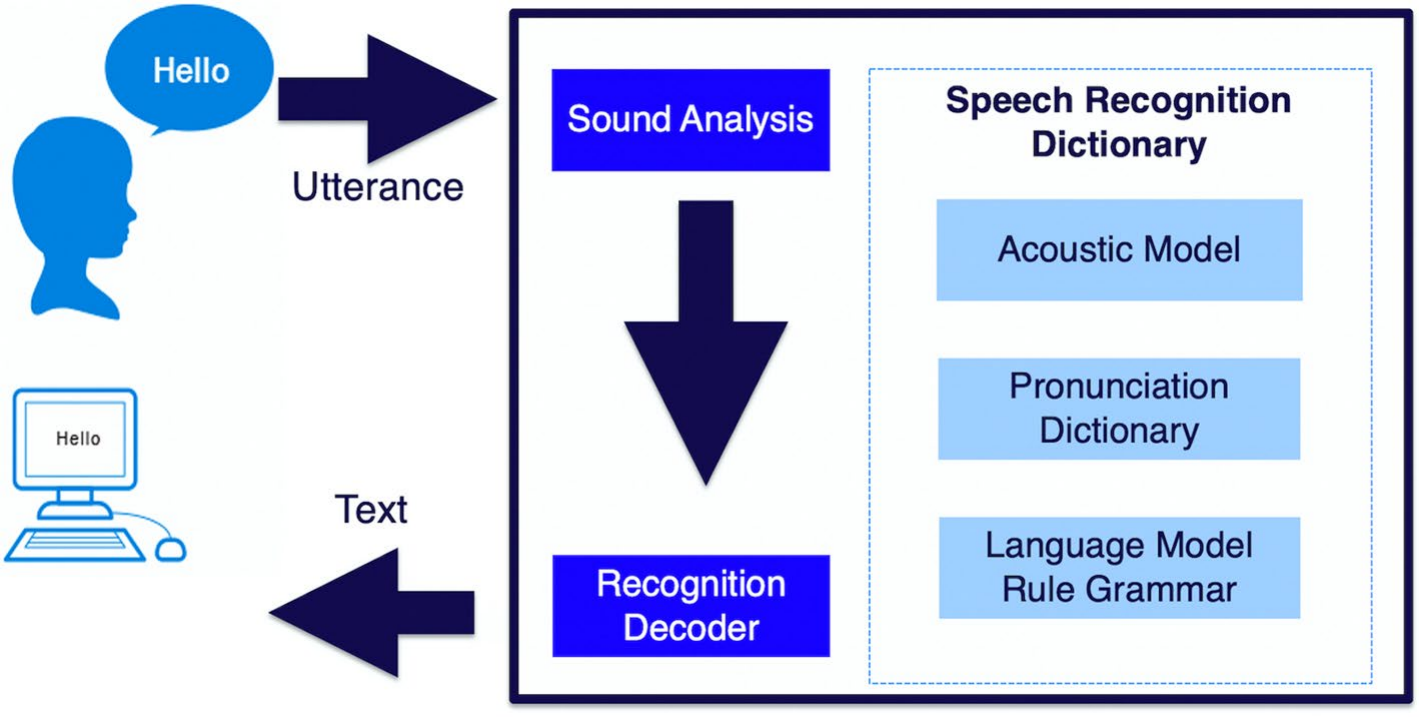
Speech recognition system. Speech recognition system *feedback method*. We recorded medical interviews using a speech recognition system and transcribed the text into Microsoft Word files

**Fig. 3:**
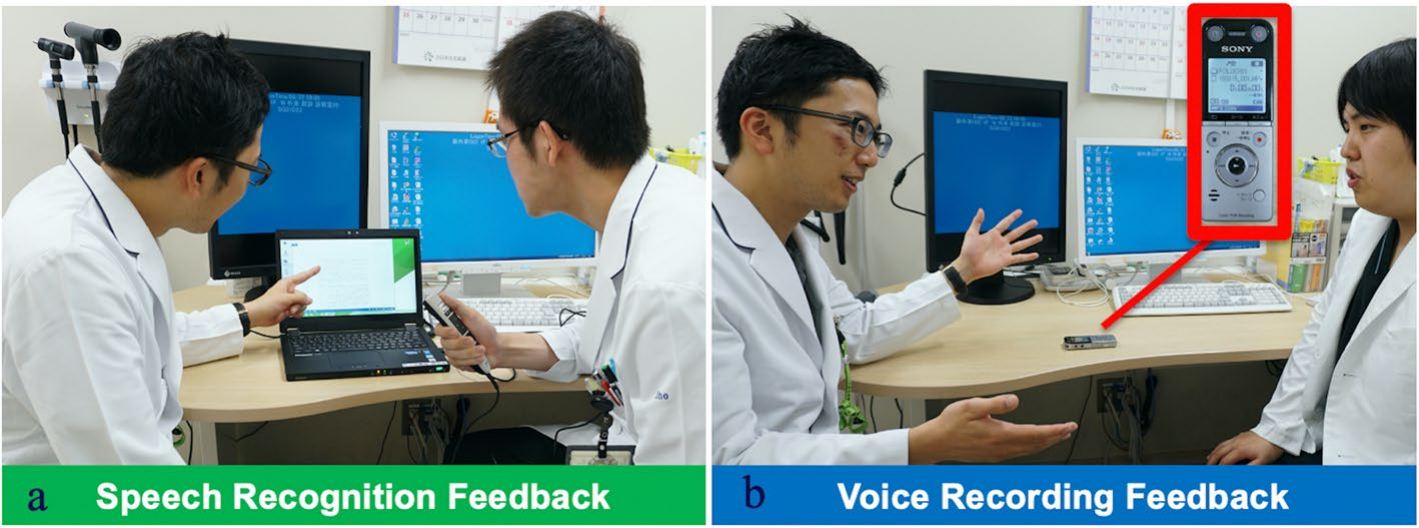
Feedback methods. **a** Speech recognition system feedback method. **b** IC recorder feedback method (control).

*SRS feedback method.* We recorded medical interviews using the SRS and transcribed the text using Microsoft Word. We used AmiVoice® Ex 7 [19] for SRS (Fig. 2). This system has a recognition rate of at least 95%, even when highly specialized medical terms are used. It uses speaker-independent technology that does not choose speakers without registration [19]. This recognition technology is not affected by intonation, accent, or speed. Faculty members used transcribed Japanese text data to provide feedback to students during their interviews (Fig. 3a). The text was read from the beginning, but feedback was provided by stopping midway and highlighting the key points. We checked the sentences before completing the mock patient encounter.

*IC recorder feedback method.* An IC recorder was installed to record mock patient encounters. Using their recorded voices, faculty members provided feedback to medical students about their encounters (Fig. 3b). The recorded voice was played from the beginning but stopped halfway to provide feedback to students on the key points. In principle, faculty members check the recorded voices before completing the mock patient encounter.

Faculty development sessions were held repeatedly through educational interventions using SRS and IC recorder feedback. Scripts were created for feedback to ensure uniform educational intervention. These feedback scripts included education on frameworks such as the PQRST (an acronym specifically for the assessment of pain) used in clinical reasoning for information gathering [20] and the pivot and cluster strategy [21], which examines analogous diseases in response to a recalled differential.

#### Second mock patient encounter

Students who received either of the two feedback types had to undergo another similar mock patient encounter a week later. Each medical student was assigned one of four prepared clinical cases (B1–B4) and asked to make another differential diagnosis based on their medical history and physical examination findings. This mock patient encounter was conducted to compare medical students’ performances between the first and second mock encounters. The evaluators in the second mock encounter were blinded to the feedback method of the student.

### Assessment of the effects of the two feedback systems

- *1). Diagnostic accuracy*
  Diagnostic accuracy was defined as the percentage of concordance of each medical student’s reported disease with a predefined final diagnosis. Medical students were asked to make the most appropriate diagnosis based on their medical history and physical examination findings during the first and second mock patient encounters. A final diagnosis was made for each case, and when the students’ diagnoses matched the diagnosis, it was judged as correct.
- *2) Mini-CEX*
  During the first and second mock patient encounters, the teachers evaluated the students’ medical interviewing skills based on the following mini-clinical evaluation exercise (mini-CEX) items [22, 23]: medical interviews, physical examinations, professionalism, organization/efficiency, and overall clinical competence. The mini-CEX was conducted by different teachers during the first and second mock encounters. These teachers, who had received training beforehand to ensure standardization of the evaluations, conducted their evaluations independently. The mini-CEX was rated on a 9-point scale, where points 1–3 represented “unsatisfactory,” 4–6 represented “satisfactory,” and 7–9 represented “beyond expectations.”
- *3). Checklists for clinical performance*
  Examinees’ performance in an objective structured clinical examination is usually assessed using checklists [24]. We developed a checklist to assess the clinical performance skills of medical students using previous research [25] and focus group discussions. The checklist included 20 items: medical interview (10 items), physical examination (5 items), and professionalism (5 items; see Supplementary 1). Each checklist element required the examiner to tick a box for the considered item.

### Evaluation of feedback efficiency

To evaluate the efficiency of the feedback methods, we measured the time spent using each feedback method in the *SRS feedback* and *IC recorder feedback* groups. The teacher used a stopwatch to measure the time from the beginning to the end of each feedback session.

#### Clinical cases

Eight test cases were identified (see Supplementary 2). Patients presented with signs and symptoms that they encountered relatively frequently in general outpatient clinics. Each scenario was based on previous research [25] and focus group discussions. These cases included depression (A1), streptococcal pharyngitis (A2), migraine (A3), carpal tunnel syndrome (A4), hypothyroidism (B1), infectious mononucleosis (B2), cluster headache (B3), and transient ischemic attack (B4). For the case scenario, four cases (A1–A4) were considered for the pre-feedback evaluation, and four cases (B1–B4) were considered for the post-feedback evaluation. These cases represent two main problems, Problems A and B, and include four main types of complaints with different final diagnoses. For specific examples, see below (listed in the order of the main complaint, final diagnosis of Problem A, and final diagnosis of Problem B):

### Statistical analysis

We compared the diagnostic accuracy in the clinical cases, mini-CEX and checklist scores, and feedback time between the groups using a paired *t*-test; we also carried out an unpaired *t*-test for post-test comparisons across the groups. Power analysis using the G*power computer program [26] indicated the need for a sample of 37 persons in each group to detect small effects (*f* = 0.25), with 80% power and alpha set at 0.05. All statistical analyses were performed using the IBM SPSS version 26.0 (IBM Corp. Armonk, NY).

### Ethics approval and consent to participate

This study was performed in accordance with the Declaration of Helsinki and was approved by the Ethics Committee and Institutional Review Board of Chiba University Graduate School of Medicine (Chiba, Japan). We explained the study to the students and obtained their informed and voluntary consent. To avoid perceived coercion, faculty members explained to students that the study would not be considered for university grading. This study was registered with the Japan Registry of Clinical Trials on July 7, 2022 (Clinical Trial Registration Number jRCT1030220188).

## Results

### Participant characteristics

All 79 participants completed the study. Their mean age was 23.6 (± 1.8) years, and 63.2% were male. No statistically significant differences could be found with regard to age and sex between the *SRS feedback* and *IC recorder feedback* groups (*p* = 0.82, 0.53, respectively).

### Outcome measures

This study focused on the differences in diagnostic accuracy and mini-CEX and checklist scores for clinical case scenarios between students enrolled in the SRS feedback method and those enrolled in the voice recording feedback method.

The post-test mean diagnostic accuracy scores were significantly higher than the pre-test scores for the *SRS feedback* group (Table 1). Post-test score comparisons between the two groups showed significant differences (*SRS feedback* group:89.7% vs. *IC recorder feedback* group:67.5%, *p* = 0.037; Table 2).

**Table 1:** Diagnostic accuracy, Mini-CEX, and checklist score on the pre-test and post-test (*n* = 79)

	**Intervention group (*n* = 39)**			**Control group (*n* = 40)**		
	**Pre test Mean**	**Post test Mean**	***p-value***	**Pre test Mean**	**Post test Mean**	***p-value***
Diagnostic accuracy, % (n)	51.3 (20/39)	89.7 (35/39)	< 0.001	57.5 (23/40)	67.5 (27/40)	0.352
Mini-CEX^a^						
Medical interviewing, (SD)	5.4 (0.2)	7.4 (0.1)	< 0.001	5.6 (0.2)	6.1 (0.2)	0.031
Physical examination, (SD)	5.0 (0.2)	6.9 (0.1)	< 0.001	4.9 (0.3)	5.7 (0.2)	0.006
Professionalism, (SD)	5.4 (0.2)	6.9 (0.1)	< 0.001	5.9 (0.2)	6.4 (0.2)	0.007
Organization / Efficiency, (SD)	5.2 (0.2)	7.2 (0.2)		5.6 (0.2)	6.1 (0.2)	0.014
Overall clinical competence, (SD)	5.2 (0.2)	7.4 (0.2)	< 0.001	5.6 (0.2)	6.1 (0.2)	0.022
Checklist score^b^						
Medical interviewing, (SD)	6.4 (0.2)	8.3 (0.2)	< 0.001	6.8 (0.2)	7.0 (0.2)	0.590
Physical examination, (SD)	2.9 (0.2)	3.7 (0.1)	0.001	2.9 (0.2)	3.5 (0.2)	0.001
Professionalism, (SD)	2.9 (0.2)	3.7 (0.2)	< 0.001	3.5 (0.2)	3.4 (0.2)	0.584
Total, (SD)	12.2 (0.4)	16.1 (0.3)	< 0.001	13.1 (0.4)	13.8 (0.4)	0.107

^a^Mini-CEX: 1 − 9

^b^Checklist: Medical interviewing: 0 − 10, Physical examination: 0 − 5, Professionalism: 0 − 5, Total: 0 − 20

**Table 2:** Diagnostic accuracy, Mini-CEX, and checklist score on the post-test (*n* = 79)

	**Intervention group (*n* = 39)**	**Control group (*n* = 40)**	***p-value***
	**Post test Mean**	**Post test Mean**	
Diagnostic accuracy, % (n)	89.7 (35/39)	67.5 (27/40)	0.037
Mini-CEX^a^			
Medical interviewing, (SD)	7.4 (0.1)	6.1 (0.2)	< 0.001
Physical examination, (SD)	6.9 (0.1)	5.7 (0.2)	< 0.001
Professionalism, (SD)	6.9 (0.1)	6.4 (0.2)	0.018
Organization / Efficiency, (SD)	7.2 (0.2)	6.1 (0.2)	< 0.001
Overall clinical competence, (SD)	7.4 (0.2)	6.1 (0.2)	< 0.001
Checklist score^b^			
Medical interviewing, (SD)	8.3 (0.2)	7.0 (0.2)	< 0.001
Physical examination, (SD)	3.7 (0.1)	3.5 (0.2)	0.367
Professionalism, (SD)	3.7 (0.2)	3.4 (0.2)	0.165
Total, (SD)	16.1 (0.3)	13.8 (0.4)	< 0.001

^a^Mini-CEX: 1 − 9

^b^Checklist: Medical interviewing: 0 − 10, Physical examination: 0 − 5, Professionalism: 0 − 5, Total: 0 − 20

The speech recognition and voice recording groups showed significant increases in mini-CEX scores, medical interviews, physical examinations, professionalism, organization/efficiency, and overall clinical competence in post-test compared to pre-test scores (Table 1). In the post-tests in the voice recording feedback group, overall clinical competence, in particular, increased from 5.2 ± 0.2 in pre-tests to 7.4 ± 0.2 in post-tests in the SRS feedback group; it increased from 5.6 ± 0.2 in pre-tests to 6.1 ± 0.2 in post-tests in the voice recording feedback group. Post-test score comparisons between the two groups showed significant differences (Table 2).

The post-test total checklist scores were significantly higher than the pre-test scores in the *SRS feedback* group (Table 1). Post-test comparisons of the total checklist scores showed significant differences across the two groups (16.1 ± 0.3 vs. 13.8 ± 0.4, *p* < 0.001; Table 2).

The time taken for feedback was significantly shorter in the *SRS feedback* group than in the *IC recorder feedback* group (22.6 ± 2.1 min vs. 27.7 ± 2.1 min, *p* = 0.04).

## Discussion

SRS-based feedback improved the diagnostic accuracy and objective assessment scales, including the mini-CEX and checklist scores. When the SRS-based feedback method is used, feedback from the teacher can be visually recognized, which is an advantage of the feedback method [9–12]. Visual recognition enables students and teachers to provide feedback to extract keywords easily. Moreover, as the entire context can be simultaneously confirmed, it is possible to simply return to the previous context, unlike in a recording. These benefits explain why SRS reduces the time required to provide feedback on medical interviews.

The mini-CEX and checklist scores showed significant improvements in the medical interviewing skills of medical students in the *SRS feedback* group (*p* < 0.001). The advantages of creating text-based medical interview content using SRS are as follows: (1) It allows for referring back and providing immediate feedback based on texts, reducing the time required; and (2) as the interview content is automatically compiled in text format, this method enhances its usability and facilitates learning. This study attempts to make the conventional medical interview feedback method more effective and easier by exploiting these advantages. Moreover, to the best of our knowledge, SRS-based feedback methods in the context of our research have not been reported in our home country or abroad so far. This can become a new strategy for graduate education in the future.

Clinical reasoning components can be sorted into seven categories: information gathering, [27, 28], hypothesis generation, [29, 30], problem representation, [28, 29], differential diagnosis, [31, 32], leading or working diagnosis, [33], diagnostic justification, [32, 34], and management and treatment [33, 35]. Clinical reasoning requires both knowledge and skill. In the pivot and cluster strategy [21], the cluster for the main complaint in the first mock interview was knowledge of the disease. However, the feedback probably did not consider that domain-specific knowledge propagation and skill improvement could improve the positive diagnostic rate. In any case, feedback emphasizing knowledge and skills can improve the rate of positive diagnoses through educational interventions.

Attitudinal factors were considered for professionalism [36]. While the mini-CEX scores showed a significant improvement in professionalism, the checklist scores did not. The mini-CEX scores were assessed using a summary evaluation. Although the items not listed on the checklist were evaluated for professionalism, significant differences were observed. However, feedback from medical interviews can improve professionalism.

### Limitations

Our study has some limitations. First, it used mock patients rather than actual patient encounters. Although the SRS-based feedback is effective in mock patient encounters, this study did not verify whether this method could be applied to actual patients. Second, the effect of educational feedback on clinical performance may depend on faculty members’ teaching skills. Note that our study designed the instructions and trained the faculty to minimize undue educational effects. Third, the software used for SRS, AmiVoice®, is available only in Japanese. Several other SRS software packages have been developed, including one in English. While some software programs incur running costs, others are free. Fourth, a problem with SRS is that speech is sometimes incorrectly transcribed. AmiVoice® Ex 7 has a recognition rate of 95% or higher, even when highly specialized medical terms are used. It has standard equipment for speaker-independent technology that does not select speakers without registration [10]. Fifth, mini-CEX scores can be accurately evaluated through multiple repetitions. In other words, the feedback must be uniform. To address this issue, a single diagnosis was established for various scenarios. It has undergone several rounds of faculty development and can be used to establish uniformity. Sixth, SRS and voice recordings cannot directly record non-verbal information. Recorded sentences and voices were used as feedback to indirectly recall students’ nonverbal performances. More robust feedback can be obtained by recording clinical situations.

## Conclusions

The study findings suggest that the SRS method allows clinical educators to better identify deficiencies in history-taking and thus enables them to provide more specific and effective feedback. SRS-based feedback improves mini-CEX scores and diagnostic accuracy while reducing the total feedback time. SRS-based feedback is an effective and efficient method for improving clinical performance.

## Supplementary Information

**Additional file 1:** **Supplementary 1.** A sample of checklist score (Case A1).

**Additional file 2:** **Supplementary 2.** Case list (Case A1–A4, B1–B4).

## Data Availability

The raw dataset supporting the conclusions of this study is available from the corresponding author on request.
