# Supplementary material for: Do clinical interview transcripts generated by speech recognition software improve clinical reasoning performance in mock patient encounters? A prospective observational study
Source: BMC Med Educ. 2023 Apr 21;23:272. doi: 10.1186/s12909-023-04246-9 (PMC10120240; doi:10.1186/s12909-023-04246-9)
Supplement: Supplementary file 1 — Additional file 1: Supplementary 1. A sample of checklist score (Case A1). [file 12909_2023_4246_MOESM1_ESM.docx]

**Supplementary 1. A sample of checklist score (Case A1)**

A1–A4 were used in the first mock doctor–patient encounter, and B1–B4 were used in the second mock doctor–patient encounter.

| Medical interviewing  (Total 10 items) | □ 1. Began the medical interview with open-ended questions. |
| --- | --- |
|  | □ 2. Asked about the time course of symptoms. |
|  | □ 3. Asked about the presence or absence of concomitant symptoms. |
|  | □ 4. Asked about depressive symptoms. |
|  | □ 5. Asked about fatigue. |
|  | □ 6. Asked about symptoms of low thyroid function (sensitivity to cold, hoarseness, constipation, dry skin). |
|  | □ 7. He was asked about the symptoms of heart failure (shortness of breath, oedema). |
|  | □ 8. Asked about symptoms of sleep apnoea (daytime sleepiness, snoring, apnoea). |
|  | □ 9. Questions were asked about medical history, alcohol use history, and smoking history (any one of them is acceptable). |
|  | □ 10 The respondents were asked about their history of taking medication. |
| Physical examination  (Total 5 items) | □ 11. Vital signs were checked. |
|  | □ 12. Palpation of the thyroid gland was performed. |
|  | □ 13 A heart murmur was confirmed. |
|  | □ 14 The tendon reflexes of the upper and lower extremities were observed. |
|  | □ 15 The oedema was confirmed. |
| Professionalism  (Total 5 items) | □ 16. Information was collected systematically. |
|  | □ 17. The atmosphere was friendly and warm. |
|  | □ 18. Made moderate eye contact and spoke with appropriate eye contact. |
|  | □ 19. Maintained a sympathetic attitude. |
|  | □ 20. They paid attention to non-verbal appeals as well as verbal ones. |
