# Supplementary material for: Do clinical interview transcripts generated by speech recognition software improve clinical reasoning performance in mock patient encounters? A prospective observational study
Source: BMC Med Educ. 2023 Apr 21;23:272. doi: 10.1186/s12909-023-04246-9 (PMC10120240; doi:10.1186/s12909-023-04246-9)
Supplement: Supplementary file 2 — Additional file 2: Supplementary 2. Case list (Case A1–A4, B1–B4). [file 12909_2023_4246_MOESM2_ESM.docx]

**Supplementary 2.** **Case list (Case A1–A4, B1–B4).**

| **(Case A1) Depression**  A 46-year-old male presented with a chief complaint of general fatigue. The malaise has persisted for 3 months, with no intra-day variation. The malaise was triggered by a car accident 3 months earlier. The patient was uninjured in the accident, but his friend was killed. He was depressed, had low motivation, sleep disturbances, and a rare death wish. No flashbacks. No snoring, no apnoea. |
| --- |
| **(Case A2) Streptococcal pharyngitis**  A 36-year-old man presented with a chief complaint of a sore throat for 2 days. His temperature was persistently in the 38℃. He had a history of contact with his children. The physical findings were enlargement of the tonsils, tongue fur, enlarged anterior cervical lymph nodes. With regard to laboratory data, tests for haemolytic streptococcus and adenovirus (rapid diagnostic test) were positive. |
| **(Case A3) Migraine**  An 18-year-old woman presented with a chief complaint of headache that had persisted since the morning. She had a throbbing headache in her left temporal region. The pain was accompanied by nausea, hyperacusis, and photosensitivity, and was exacerbated by motion, but she did not have an aura. On physical examination, her blood pressure and body temperature were normal, as were the neurological findings. Blood tests and cerebrospinal fluid examination revealed no abnormalities, and a CT scan of the head was also normal. |
| **(Case A4) Carpal tunnel syndrome**  A 50-year-old woman presented with numbness of the left hand that had persisted since the morning of the same day. She had occasionally noted similar numbness in the morning before. Physical findings included mildly abnormal sensation of the first three digits and the radial half of the fourth digit of the left hand. Tinel’s sign and Phalen’s sign were positive, while the neck compression test was negative. No neurological abnormalities were noted in the face or lower extremities and cranial nerve findings and tendon reflexes were normal. Blood tests revealed no abnormalities, while a cervical spine X-ray film, head CT scan, and head MRI were all normal. A nerve conduction velocity study revealed prolonged latency of the median nerve compound muscle action potential and delayed sensory nerve conduction. |
| **(Case B1) Hypothyroidism**  A 76-year-old woman presented with a chief complaint of malaise for 3 months. She had gained weight, but did not have exertional dyspnoea. The physical findings consisted of thinning of the eyebrows, an enlarged thyroid gland, and nonpitting oedema of the legs. The chest X-ray film showed no abnormalities and laboratory tests revealed no inflammation. Her haematology, renal function, and hepatic function tests were normal, but the level of thyroid stimulating hormone (TSH) was high. |
| **(Case B2) Infectious mononucleosis**  A 19-year-old man presented with a chief complaint of a sore throat for 10 days. His temperature was persistently in the 37.5 ℃. He had no history of contact with children. The physical findings were enlargement of the tonsils, tongue fur, enlarged posterior cervical lymph nodes, and mild splenomegaly. With regard to laboratory data, tests for haemolytic streptococcus and adenovirus (rapid diagnostic test) were negative, but he had mild hepatic dysfunction and atypical lymphocytes. Viral antibody tests revealed acute infection with the Epstein–Barr virus (EBV), while there was no evidence of human immunodeficiency virus or cytomegalovirus (CMV) infection. |
| **(Case B3) Cluster headache**  A 38-year-old man presented with a chief complaint of headache that had persisted for 180 minutes the night before. Now, he has no headache. For a week, he had a headache and cancer at night. He was in so much pain that he was sprawled out. It started at night and spontaneously lightened within 180 minutes. Strong throbbing pain on the right side of the head and behind the eyes. No sound sensitivity, no light sensitivity, no aggravation by physical movement. Tears streaming, bloodshot, nasal discharge, droopy eyelids (right eye only). |
| **(Case B4) Transient ischemic attack**  A 68-year-old man came to the hospital complaining of numbness and weakness in his right hand. About 20 minutes in the afternoon, his right hand suddenly became numb and difficult to apply force with. The onset of the disease was sudden at 12 a.m. on the same day, but he got well in 20 minutes and was symptom-free now. He had the same symptoms a month before. There was no radiation to the neck or arm. Difficulty in getting strength in the right hand. There was dysarthria, numbness in the right face, and no sensation in the lower limbs.  History of hypertension, dyslipidaemia, and diabetes mellitus. Tobacco 20 cigarettes per day x 48 years, drinking 500mL beer. |
